# What is the appropriate “first lymph node” in the era of segmentectomy for non-small cell lung cancer?

**DOI:** 10.3389/fonc.2022.1078606

**Published:** 2023-01-26

**Authors:** Joseph Seitlinger, Florent Stasiak, Juliette Piccoli, Gabriele Maffeis, Arthur Streit, Christophe Wollbrett, Joelle Siat, Guillaume Gauchotte, Stéphane Renaud

**Affiliations:** ^1^ Thoracic Surgery Department, University Hospital Nancy, Nancy, France; ^2^ Pathology Department, University Hospital Nancy, Nancy, France; ^3^ Research Unit, INSERM, Nancy, France

**Keywords:** sentinel lymph node, lung cancer, segmentectomy, fluorescence guided surgery, electromagnetic navigational bronchoscopy, lung resection

## Abstract

**Introduction:**

The place of segmentectomy in the management of lung cancer is shifting following the inspiring results of the Japanese JCOG0802 trial. I n this study, authors suggested that performing segmentectomy would require in an optimal way an intraoperative confirmation of pN0 tumor with a frozen section. Our objective was to determine whether the proposed technique, i.e. adjacent lymph node analysis, is consistent with the results of our study on sentinel lymph node (SLN) detection using fluorescence.

**Methods:**

This is a retrospective, observational, single center study. Eighty-one patients with suspected localized stage NSCLC (IA to IIA) were included between December 2020 and March 2022. All patients received an intra-operative injection of indocyanine green (ICG) directly in the peritumoral area or by electromagnetic navigational bronchoscopy (ENB). The SLN was then assessed by using an infrared fluorescence camera.

**Results:**

In our cohort, SLN was identified in 60/81 patients (74.1%). In 15/60 patients with identified SLN (25%), NIR-guided SLN was concordant with the suggestions of JCOG0802 study. A retrospective SLN pathological analysis was performed in 43 patients/60 cases with identified SLN (71.2%), including 37 cases of malignant disease. Occult micro-metastases were found in 4 patients out of 37 SLN analyzed, leading to a 10.8% upstaging with NIR-guided SLN analysis.

**Dicussion:**

At the time of segmentectomies, ICG technique allowed the identification of the SLN in a high percent of cases and in some areas usually out of the recommended stations for lymph node dissection.

## 1 Introduction

Lung cancer remains the leading cause of cancer-related deaths worldwide, responsible for more than 1.7 million deaths per year (1). For early stages, the cornerstone of treatment remains surgery. Surgical resection of lung cancer must meet a certain number of oncological criteria such as free resection margins and completion of a systematic lymph node dissection. It must include, at least, three nodes removed from both pulmonary and mediastinal stations, including station 7 (subcarinal) which is mandatory (2). Meanwhile lobectomy has been the gold standard of care for decades, segmentectomy has recently gained interest in major lung resections (3).

The place of segmentectomy is now well codified by recommendations of scientific societies, such as the ERS/ESTS (4, 5), This kind of lung resection can be offered for lung tumors measuring less than 2 cm, without lymph node involvement on preoperative examinations in patients with impaired respiratory function. In recent years, these lines have tended to move, notably thanks to several Japanese studies (JCOG 1211/JCOG 0802) (6). Although local control seems to be inferior to lobectomy in these selected patients whose respiratory function would allow segmentectomy or lobectomy, overall survival was better in the segmentectomy group compared to lobectomy. However, this trial included only large-volume Japanese centers, which have extensive experience with segmentectomy.

One of the principles of segmentectomy in JCOG 0802 study is to perform an intraoperative confirmation of eligibility before to proceed to segmentectomy. This intra-operative confirmation relies on frozen section analysis of the removed adjacent lymph node. If the frozen section analysis is positive, the segmentectomy procedure is converted to lobectomy. The question of the lymphatic draining system from the tumor is therefore central, and several issues arise. In this study, the lymph node located at the foot of the segmental bronchus (adjacent lymph node) was systematically sent for frozen section. However, due to aberrant lymphatic drainage paths in the thorax, the first lymph node relay may be located at different lymph node station. Indeed, there may be a direct mediastinal involvement such as well-described skip N2 (7), a hilar or scissural involvement, adjacent lymph node involvement or even an isolated lymph node involvement (8).

To assess the relevance of lymph node analysis as described in the JCOG 0802 study, we retrospectively analyzed our lymphatic drainage data of Near InfraRed (NIR)-guided sentinel lymph node detection during major lung resection surgery.

The main objective of our study was to determine whether the NIR-guided sentinel lymph node (SLN) technique during major lung resection demonstrates good concordance with those recommendations. This question would bring back into the debate the place of the sentinel lymph node technique in the era of cancer segmentectomies.

## 2 Methods

### 2.1 Ethical statement

This study was approved by our Institutional Review Board. Patients retrospectively gave consent to use their data or did not respond that they objected within 2 months.

### 2.2 Statistical analysis

Independent binary proportions or categorized variables were compared *via* chi-squared testing using Stata software. Descriptive characteristics were produced including median, interquartile rang for continuous parameters and frequency distributions (number and proportion) for categorical parameters were produced for all patient demographics and baseline characteristics.

### 2.3 Study design

This is a retrospective, observational, single-center trial, performed in the thoracic surgery department of the Regional University Hospital of Nancy (France).

We collected data from patients who underwent a major lung resection (i.e. lobectomy and segmentectomy) for suspected NSCLC or proved NSCLC cT1a-T2bN0 between December 2020 to March 2022.

All included patients benefited from a per-operative injection of indocyanine green for sentinel lymph node (SLN) assessment. Pre-operative staging included chest and abdominal CT-scan, as well as a PET-scan. Brain imaging was performed by MRI or CT-scan depending on availability of the exam.

Patients were operated either by open thoracotomy, video-assisted thoracoscopic surgery (VATS) or robot-assisted thoracoscopic surgery (RATS), according to surgeon preference. A radical lymph node dissection was routinely performed as recommended (4).

### 2.4 Study endpoints

The primary endpoint of this study was the accuracy between the identification of the sentinel node by NIR fluorescence during major lung resection and the JCOG 0802 study recommendations.

The location of the detected sentinel lymph node was considered compatible with the JCOG0802 study recommendations when the sentinel lymph node was at station 11 in the case of lobectomy or at station 12 or 13 in the case of segmentectomy.

The secondary endpoints were the identification rate of one or more sentinel lymph node(s) by NIR fluorescence imaging after transpleural or transbronchial injection of ICG and the microscopic lymph node invasion (LNI).

In addition, adverse effects related to ICG or injection techniques have been collected.

### 2.5 Intraoperative technique

Injection method of ICG was performed as previously published by Philips (9). In summary, a peri-tumoral injection of 1mL of ICG, was performed by transpleural or transbronchial method.

#### 2.5.1 Transpleural injection

In cases of direct transpleural approach, ICG was injected through the incision by a 19G fine needle (Arcpoint ^®^, Medtronic) into the peri-tumoral area, at a depth of at least 1cm in the parenchyma to limit diffusion of ICG in the chest cavity.

#### 2.5.2 Transbronchial injection

Bronchoscopic navigation in the airways was performed by ENB, using the Illumisite ^®^ platform from Medtronic (Minneapolis, Minnesota) as previously described (9).

Once near the lesion, a 19G needle (Arcpoint ^®^, Medtronic) was inserted through the catheter and injection of ICG was performed.

The assessment of the SLN by a NIR camera (Visionsense^©^, Medtronic) was initiated after at least 5 minutes of bi-pulmonary ventilation. A lymphatic mapping by NIR camera was then performed. If a SLN was fluorescent, it was resected and a systematic lymph node dissection was performed in all cases as recommended. A specific mode of the NIR camera called “tumor mode” was used during lymph node mapping.

Once resected, the fluorescent lymph node was sent to the department of pathology apart from the other lymph nodes in order to perform specific IHC analysis with the anti-cytokeratin antibody AE1/AE3.

## 3 Results

### 3.1 Cohort characteristics

According to our inclusion criteria, 81 patients were included. The median age at surgery was 68.5 years (IQR=12). The male to female ratio was 0.59 for men. The proportion of lobectomy and segmentectomy was 51.8% and 48.2% respectively (**Table 1**).

**Table 1:** Patients characteristics (n=81).

Variable		P
Age	68 (IQR 12)	
Sex Male Female	47 (58) 34 (42)	
Body mass index	27.6 (IQR 6)	
Performance status	1 (IQR 0)	
Charlson Comorbidity Index	5 (IQR 2)	
Comorbidities Cardiac (1) (n=81) Vascular (2) (n=81) Respiratory (3) (n=81) Digestive pathology (4) (n=81) Neurology (5) (n=81) Cancer history (6) (n=81) Hematology (7) (n=81) Nephrology (9) (n=81) Endocrine disease (n=70)	53 (65.4) 21 (25.9) 38 (46.9) 18 (22.2) 11 (13.5) 34 (41.9) 8 (9.8) 7 (8.6) 14 (20)	
Preoperative FEV1* DLCO*	86 (IQR 34) 75 (IQR 24)	
Smoking history (n=80) Pack years	62 (77.5) 30 (IQR 20)	
Type of surgery Lobectomy (n=81) Segmentectomy (n=81)	42 (51.8) 39 (48.2)	
Method of injection ENB injection (n=57) Direct Injection (n=24) Overall operating time Operating time (ENB technique) Operating time (direct injection technique) ENB injection Transbronchial ICG injection time Mapping of SLN time Direct injection Direct injection, 10 min. ventilation and mapping of SLN time	57 (70.3) 24 (29.7) 92.5 (IQR 70) 90 (IQR 50) 130 (IQR 85) 10 (IQR 5) 13 (IQR 11) 23 (IQR 5)	0.09
SLN identification ENB (n=57) Direct injection (n=24) Mono-site (ENB) Multi-site (ENB) Mono-site (Direct) Multi-site (Direct)	43 (75.4) 17 (70.8) 42 (97.6) 1 (2.4) 12 (70.6) 5 (29.4)	0.74 0.01

A minimally invasive approach (VATS and RATS) was mainly performed (74/81, 91.4%). cTNM and pTNM distribution of the included patients is presented in **Table 2**.

**Table 2:** cTNM and pTNM Stage.

cTNM Stage (8^th^ TNM)	IA1	IA2	IA3	IB	IIA		
N (%) (n=81)	18 (22.2)	35 (43.2)	12 (14.8)	11 (13.6)	5 (6.2)		
pTNM Stage (8^th^ TNM)	IA1	IA2	IA3	IB	IIA	IIB	IIIA
N (%) (n=56)	7 (12.5)	19 (33.9)	13 (23.2)	12 (21.4)	1 (1.8)	3 (5.4)	2 (3.6)

Median number of removed lymph node was 10 (IQR=11).

Median time for ICG transbronchial injection was 10 minutes (IQR=8). There was no complication due to ICG injection. Median time of surgery was 95 min (IQR=70), including 15 minutes (IQR=11) dedicated to mapping of sentinel lymph node.

There were 64 malignant lesions in the 81 patients included (79%): 39 adenocarcinomas, 15 squamous cell carcinomas, 2 small cell neuroendocrine carcinoma, 1 mixed carcinoma (adenocarcinoma + small cells), 3 metastases of different origin (breast, endometrium, prostate), 3 carcinoid tumors, 1 of which was atypical, and 1 plasmacytic granuloma.

### 3.2 Sentinel lymph node feasibility

**Figure 1:**
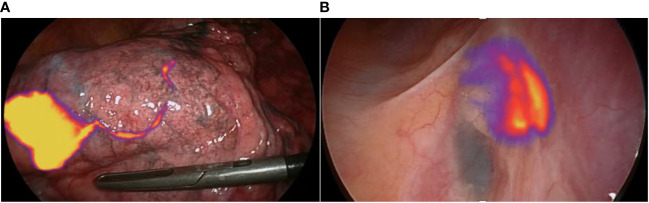
NIR-Guided sentinel lymph node specificity: **(A)** The lymphatic pathway is clearly seen with NIR on the surface of the lung, starting from the lung nodule located in the right lower lobe here. **(B)** The intraoperative view shows fixation at station 7. Only one of the two lymph nodes is intensely fixed with the NIR-camera in “Tumor” mode: this is the sentinel lymph node.

In total, 60 sentinel lymph nodes were identified with NIR-guided fluorescence imaging (60/81, 74.1%) (**Figure 1**). Sentinel lymph node identification was possible in 17/24 patients (70.9%) in the direct injection group and in 43/57 patients (75.4%) in the ENB injection group. There was no significant difference between the groups (p=0.74).

Regarding the 21 cases of SLN identification failures, there were 6 cases with significant anthracosis limiting the detection of fluorescence (28.6%), 5 technical problems (camera) (23.8%), 4 cases with no marked nodule (related to injection issue) (19%), and 6 cases with a marked nodule but no discernible path towards a lymph node (28.6%).

### 3.3 Accuracy of lymph node analysis during major lung resection

About the 60 identified SLN, 15 SLN (25%) would have been collected and sent for frozen section analysis according to JCOG0802 study suggestions. If only lung cancers with identified SLN were considered, 11/47 (23.4%) would have been consistent with the JCOG0802 study suggestions.

Concerning the type of resection performed, there was a statistically significant difference in the accuracy of the SLN location (compared to JCOG0802) between lobectomy (14/33, 42.4%) and segmentectomy (1/27, 3.7%) (p<0.001) (**Table 3**).

**Table 3:** Accuracy of lymph node analysis.

Type of surgery	Accuracy with adjcent lymph node	p=
Lobectomy with SLN identified (%)(n=33)	14 (42.4)	p<0.001
Segmentectomy with SLN identified (%) (n=27)	1 (3.7)	

### 3.4 Sentinel lymph node location

There was a unique identification of the sentinel node in 54/60 patients (90%).

Injection was performed by direct route in 24 patients (29.6%) and by ENB in 57 patients (70.4%). In direct injection group, the sentinel node was identified in 17 cases out of 24, including 13 mono-site (76.5%) and 4 bi-site (23.5%). With ENB injection, the sentinel node was identified in 43 patients including 1 bi-site (2.3%). There was a statistically significant difference in the detection of bi-site or mono-site SLN according to the injection technique (ENB injection vs direct injection). (p=0.01).

Additional data regarding the type of resection performed, the location of the nodule at the segmental level as well as the SLN identified are available in a table in the **Supplementary Data**.

**Figure 2:**
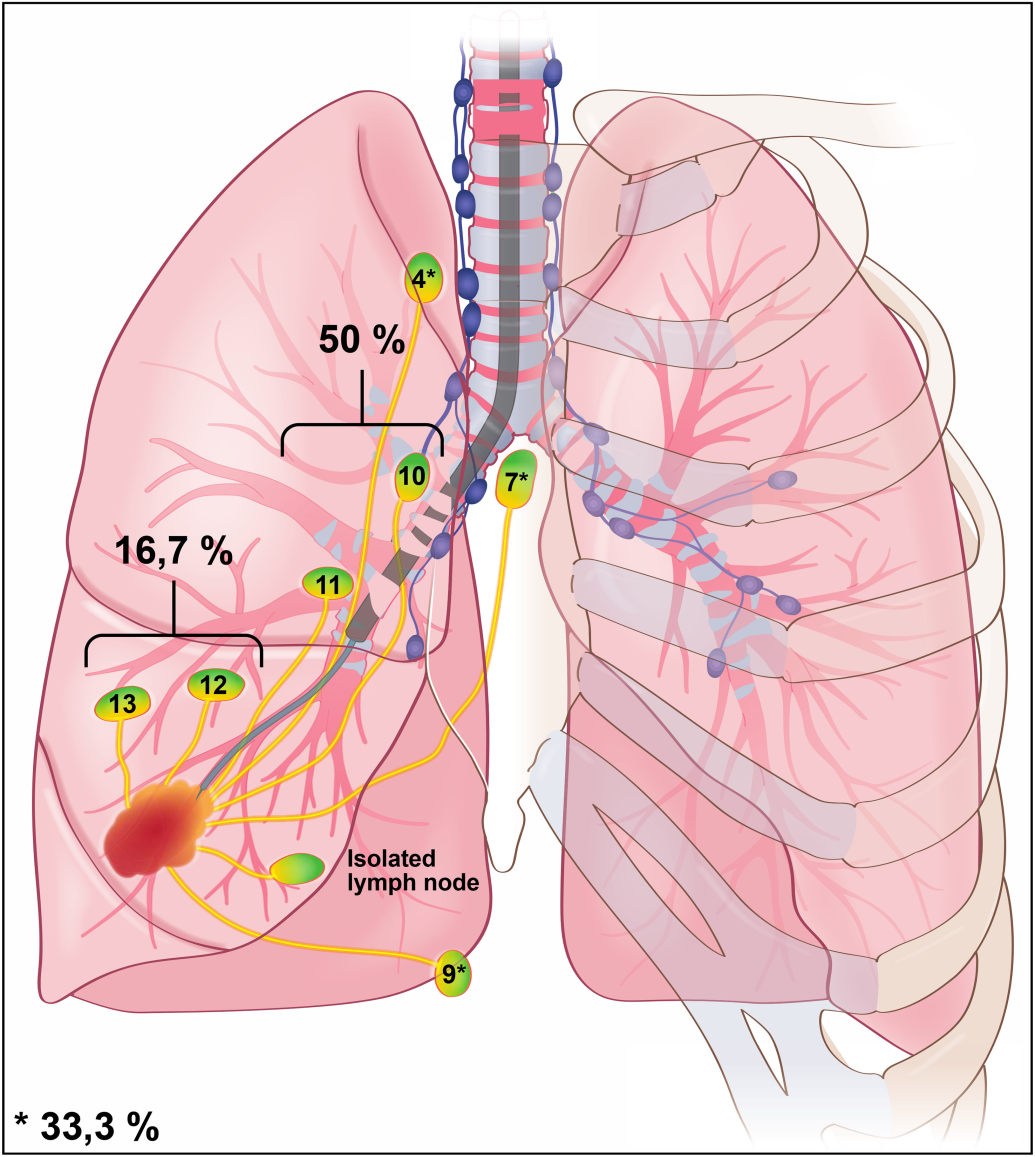
Illustration of peri-tumoral ENB ICG injection. Disclosed percentages represent data for the whole cohort (regardless the location of the tumor). The percent of cases according to the location (mediastinal, hilar, scissural or iLN) is specified (in the majority of cases the lymph node is single (90%). 18/54 mediastinal (33.3%), 27/54 hilar (50%), 9/54 scissural and iLN (16.7%).

Considering only the location of the single-site lesions, we found 18/54 (33.3%) mediastinal “skip N2” SLN, 27/54 (50%) hilar SLN, 4/54 scissural SLN (7.4%), and 5/54 (9.3%) so-called “isolated” SLN (**Figure 2**).

### 3.5 Sentinel lymph node invasion

Of the 60 patients in whom the sentinel node was identified, there were 14 diagnoses of benign lesions for which pathological analysis was not performed. Of the 46 malignant lesions with sentinel lymph node identification, the sentinel node was examined in 37 cases (5 cases of “isolated” intra-parenchymal nodes, not removed, 3 with no pathological examination, 1 without lymph node in specimen).

**Figure 3:**
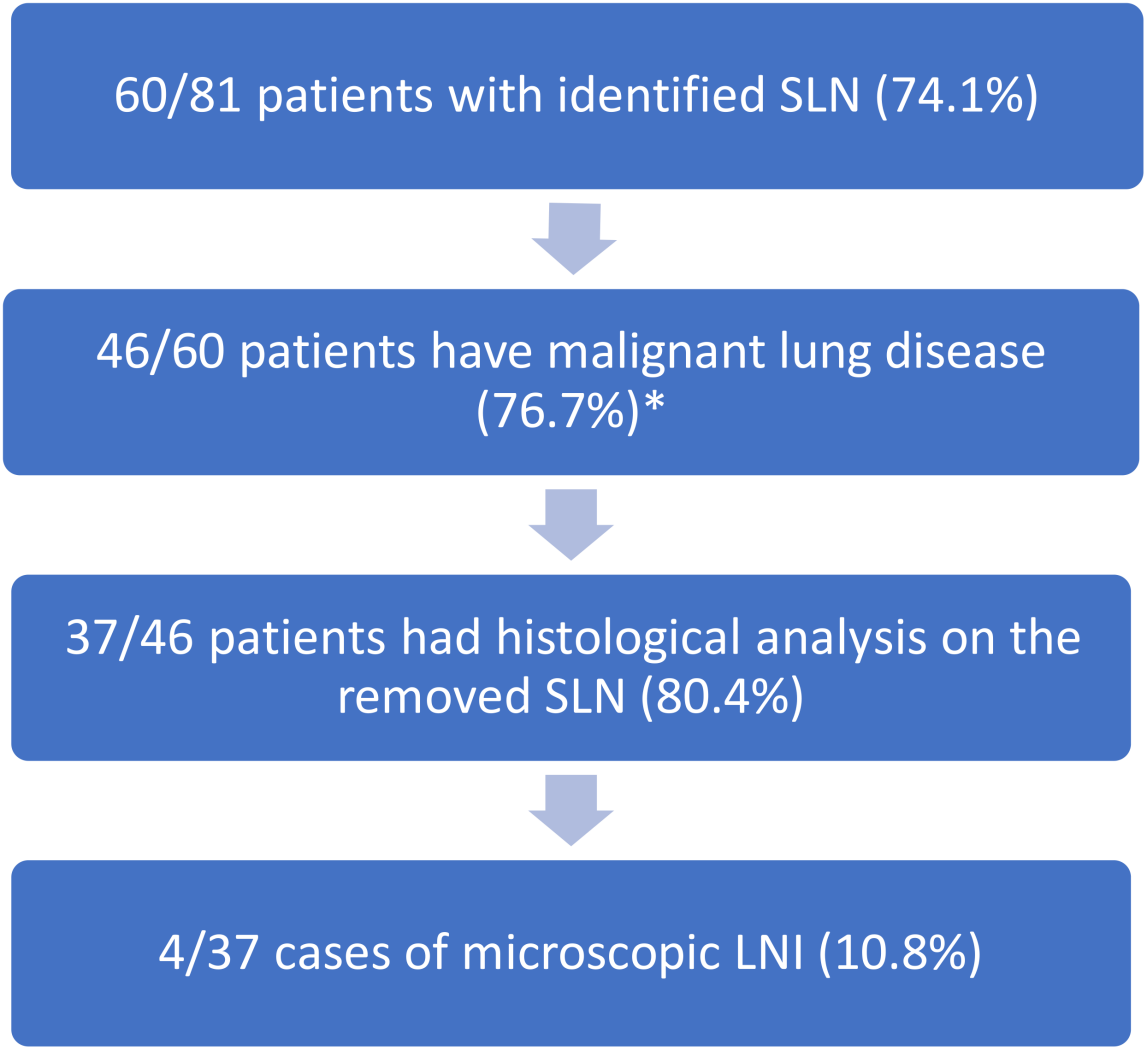
Flow chart of the 81 patients included in the study. *: 3 patients with metastases were removed from this analyze.

In total, 4 cases of submilimetric lymph node invasion (occult micro-metastasis) were identified in 37 patients (10.8%). For the 33 other patients with negative sentinel lymph nodes, the final analysis of the radical lymph node dissection showed only pN0 patients (100%) (Flow Chart, **Figure 3**).

In addition, in 4 patients with microscopic invasion of the sentinel lymph node, we performed additional immunohistochemical analysis in thin sections on the other lymph nodes without finding any other occult microscopic metastasis.

## 4 Discussion

The place of the sentinel lymph node in lung cancer surgery remains unclear.

At the time of segmentectomies, the JCOG0802 study has shown very interesting results which should modify our practice. In this study, the authors propose an intraoperative confirmation of eligibility for segmentectomy which consist of frozen section analysis of the so called “adjacent lymph node”. In our cohort, the degree of concordance between this lymph node and the lymph node indicated by the diffusion of indocyanine green was low (25%). To our knowledge, this is the largest cohort on sentinel lymph node technique in the field of thoracic surgery.

The discrepancy between the sentinel lymph node removed after ICG injection and the adjacent lymph node removed in the JCOG 0802 settings puts the sentinel lymph node at the center of the debate. Indeed, in our study, in 75% of cases, the lymph node removed according to the JCOG0802 suggestions does not appear to be the first lymph node relay. Moreover, this idea is reinforced by the fact that none of the positive lymph nodes was an adjacent lymph node: two were hilar and the two others were mediastinal lymph nodes.

This differential can probably be explained by different factors: the high proportion of N2 skips (33.3%) which is in conformity with the published data (7), and the frequency of intra-parenchymal nodes or “isolated” lymph nodes (iLN). Isolated lymph node represents nearly 10% of the sentinel lymph nodes identified and raise the problem of their surgical removal and analysis. Furthermore, the prevalence of this isolated lymph node (10%), which is not removed during segmentectomy, may partly explain the higher local recurrence rate demonstrated in the JCOG0802 study for segmentectomy.

The limits of our study are the risk of downstaging in patients whose sentinel lymph node had not been removed intraoperatively, e.g., in the case of intra parenchymal lymph node (“isolated lymph node”). Furthermore, it is a single center study with a limited number of patients enrolled. However, there are presently few centers equipped with ENB system (or other advanced fibroscopic system) and a NIR-camera, but these results could accelerate their acquisition by other thoracic surgery departments. Similarly, the presence of a NIR-camera on the Da Vinci^©^ robot system (Intuitive SA) could allow this technique to be performed in many centers.

The strong result of our study is obviously the fact that there were 100% pN0 patients in patients with negative SLN. The only lymph nodes that were positive were the SLN with microscopic involvement (4/37 cases). Moreover, this technique allowed us to highlight 4 micro-metastases that would not have been visualized in standard pathology (10.8% lymph node upstaging). This data reinforces the idea that this sentinel lymph node technique could identify occult micro metastases and improve staging.

The impact of these micro-metastases remains poorly understood in lung cancer. Analysis of survival data (OS/DFS) of SLN-negative patients (confirmed pN0 patients) compared to pN0 patients without sentinel lymph node mapping will allow us to evaluate the prognostic impact of this procedure. These data should be the subject of a future publication.

**Figure 4:**
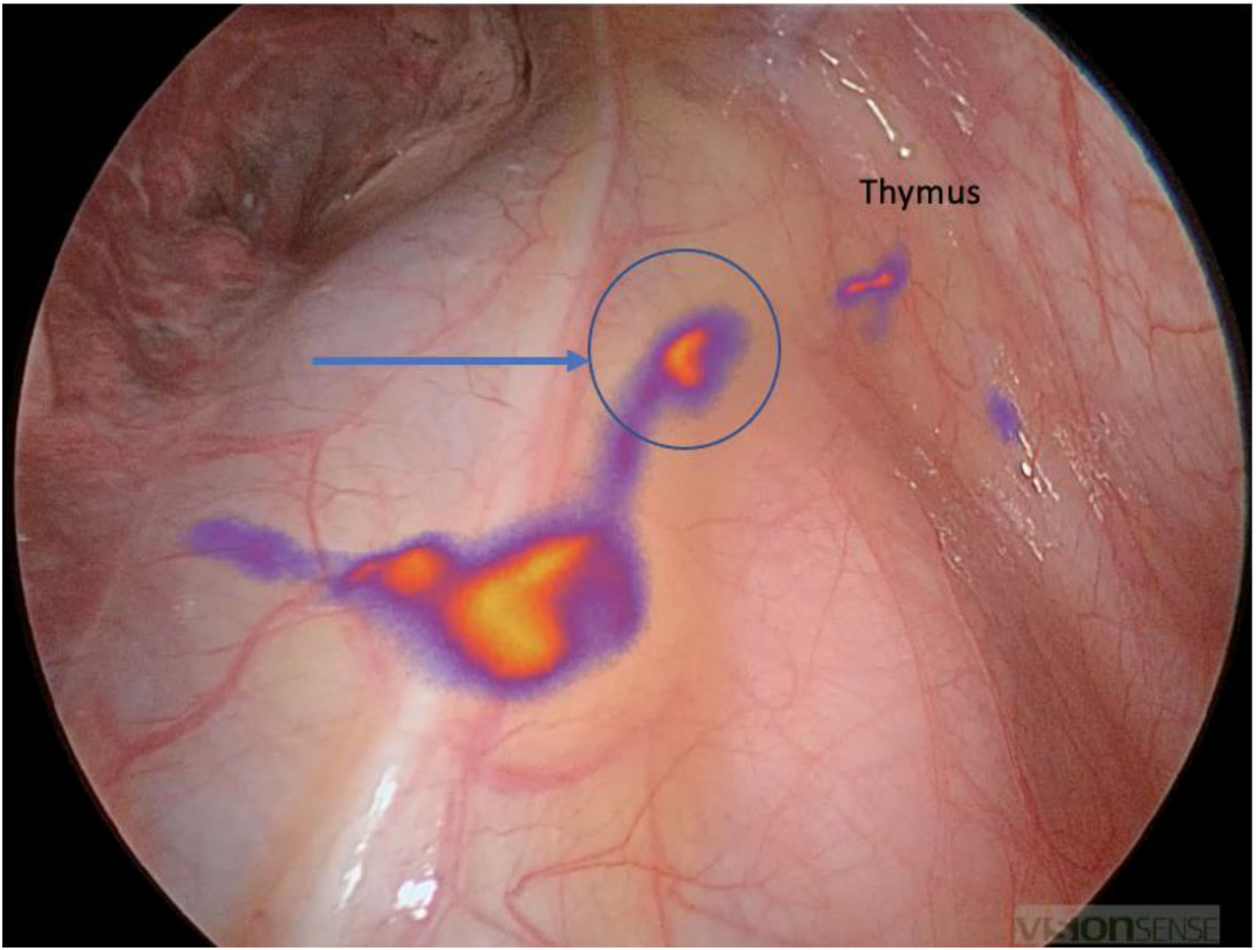
Atypical location of a sentinel lymph node in position 3A in the context of a right S6 segmentectomy: the path is seen in front of the superior vena cava and then a fixation at the lymph node level in front of the right phrenic nerve (blue arrow). Note the fixation of the thymus on the right upper part of the image.

In addition, the sentinel lymph node mapping technique by fluorescence permitted us to identify sentinel lymph nodes located outside of the most performed lymph node dissection sites (**Figure 4**).

At the time of segmentectomies, ICG technique allowed the identification of the SLN in a high percent of cases and in some areas usually out of the recommended sites for lymph node dissection. These results could change the practice of segmentectomy in the future with systematic intraoperative confirmation by frozen section, guided by a successful identification of the sentinel lymph node. Finally, in the era of immunotherapy, the performance of a systematic lymph node dissection in patients whose sentinel lymph node is not invaded (confirmed with frozen analysis) must be put into perspective in view of the possibility of peri-operative effective immunotherapy treatment (10).

## Data availability statement

The raw data supporting the conclusions of this article will be made available by the authors, without undue reservation.

## Ethics statement

The studies involving human participants were reviewed and approved by Institutional Review Board, University Hospital Nancy. The patients/participants provided their written informed consent to participate in this study.

## Author contributions

JSe, FS, GM, AS, CW contributed to the collection of patients’ clinical data. GG contributed to the samples analysis. JS, SR, JSi, GG contributed to the design of study, to the analysis of the results and to the writing of the manuscript. All authors contributed to the article and approved the submitted version.
