# Supplementary material for: What is the appropriate “first lymph node” in the era of segmentectomy for non-small cell lung cancer?
Source: Front Oncol. 2023 Jan 26;12:1078606. doi: 10.3389/fonc.2022.1078606 (PMC9909202; doi:10.3389/fonc.2022.1078606)
Supplement: Supplementary file 1 [file Table_1.pdf]

Table 1 : Type of resection, segment location and histology for all patients with identified ICG-guided Sentinel Lymph Node

Patients	Extent of resection	Tumor Segment	Lobe	Histology	SLN
1	S2 D	S2	RUL	Atypic adenomatous hyperplasia	4R
2	S6 G	S6	LLL	Chondroma	11L
3	LID	S9	RLL	Hemangioma	12R
4	S6 D	S6	RLL	AD	4R
5	S1 S2 S3 G	S1 S2	LUL	AD	10L
6	S3 D	S3	RUL	SCC	10R
7	S1 D	S1	RUL	AD	7
8	S1 S2 G	S1 S2	LUL	AD	10L
9	S1 S2 S3 G	S3	LUL	Carcinoid tumor	10L
10	S1 D	S1	RUL	SCC	11R
11	S1 S2 G	S1 S2	LUL	AD	5L
12	S1 S2 G	S1 S2	LUL	AD	10L

13	S1 S2 G	S1 S2	LUL	AD	10L
14	S1 S2 G	S1 S2	LUL	SCC	11L
15	S1 S2 S3 G	S3	LUL	ADK	11L
16	S1 S2 S3 G	S3	LUL	Metastasis	10L
17	S1 S2 S3 G	S3	LUL	SCLC	11L
18	LID	S7	RLL	Carcinoid tumor	7
19	LSD	S3	RUL	Carcinoid tumor	4R+7
20	LSD	S3	RUL	SCC	4R
21	LSD	Central	RUL	AD	10R
22	LSG	S1 S2	LUL	AD	5L
23	LID	S8	RLL	AD	7
24	LSD	S1	RUL	AD	10R
25	LSG	S1 S2	LUL	AD	5L

26	LSD	S1	RUL	AD	12R
27	LSD	S2	RUL	AD	7
28	LID	S8	RLL	AD	9R
29	LIG	S9-S10	LLL	AD	7+ 10L
30	S1 S2 G	S1-S2	LUL	Benign	10L
31	S1 S2 S3 G	S3	LUL	Atypic Hyperplasia	10L
32	LSD	S1	RUL	Bronchiolitis	7
33	LSD	S2	RUL	Pneumonia with granulomatosis	7
34	LSG	S1-S3	LUL	Granulomatosis	5L
35	S1 S2 G	S1-S2	LUL	AD	5L
36	S1 D	S1	RUL	AD	IP
37	S1 S2 G	S1-S2	LUL	SCC	5L
38	S2 D	S2	RUL	AD	10R
39	S1 S2 G	S1-S2	LUL	AS	IP

40	LSD	S1-S2	RUL	AD	IP
41	LSD	S2	RUL	SCC	IP
42	LSD	S2	RUL	AD	IP
43	S1 S2 G	S1-S2	LUL	Fibrosis	5L
44	S1 D	S1	RUL	Granulomatosis with necrosis	4R
45	S6 D	S6	RLL	AD	7 +3AR
46	LM	S4	ML	Macrophagic alveolitis	11R
47	LID	S6	RLL	Pneumonia	10+11R
48	S6 G	S6	LLL	Metastasis	12L
49	LIG	S6	LLL	AD	11L
50	LID	S9	RLL	SCC	11R
51	LM	S4	ML	AD	11R

52	LID	S10	RLL	SCC	11R
53	LID	S6	RLL	AD	11R
54	LID	S6	RLL	AD	11R
55	LSD	S1	RUL	AD	11R
56	LSD	S1	RUL	SCLC	11R
57	LIG	S10	LLL	AD	11L
58	LM	S5	ML	Plasmocytic granuloma	11+12L
59	LM	S5	ML	Fibrosis	11R
60	LSD	S2	RUL	Bronchiectasis	11R
